# A Simple-to-Perform *ifn-γ* mRNA Gene Expression Assay on Whole Blood Accurately Appraises Varicella Zoster Virus-Specific Cell-Mediated Immunity After Allogeneic Hematopoietic Stem Cell Transplantation

**DOI:** 10.3389/fimmu.2022.919806

**Published:** 2022-07-27

**Authors:** Mathilde Boccard, Anne Conrad, William Mouton, Florent Valour, Chantal Roure-Sobas, Emilie Frobert, Barbara Rohmer, Vincent Alcazer, Hélène Labussière-Wallet, Hervé Ghesquières, Fabienne Venet, Karen Brengel-Pesce, Sophie Trouillet-Assant, Florence Ader

**Affiliations:** ^1^ Centre International de Recherche en Infectiologie (CIRI), Inserm U1111, Université Claude Bernard Lyon 1, CNRS, UMR5308, Ecole Normale Supérieure de Lyon, Univ Lyon, Lyon, France; ^2^ Département des Maladies infectieuses et tropicales, Hôpital de la Croix-Rousse, Hospices Civils de Lyon, Lyon, France; ^3^ Laboratoire de Recherche Commun (LCR), Hospices Civils de Lyon/BioMérieux, Pierre-Bénite, France; ^4^ Institut des Agents Infectieux, Hôpital de la Croix-Rousse, Hospices Civils de Lyon, Lyon, France; ^5^ Service d’Hépatologie Gastro-Entérologie et Nutrition Pédiatriques, Hôpital Femme Mère Enfant, Hospices Civils de Lyon, Bron, France; ^6^ Département d’Hématologie clinique, Centre Hospitalier Lyon Sud, Hospices Civils de Lyon, Pierre-Bénite, France; ^7^ Laboratoire d’Immunologie, Hospices Civils de Lyon, Lyon, France; ^8^ EA7426 UCBL1-HCL-bioMérieux Pathophysiology of Injury-induced Immunosuppression, Lyon, France

**Keywords:** varicella zoster virus, hematopoietic stem cell transplantation, cell-mediated immunity, immune functional assays, interferon gamma, antiviral prophylaxis

## Abstract

Herpes zoster, which is due to the reactivation of Varicella zoster virus (VZV), is a leading cause of morbidity after allogeneic hematopoietic stem cell transplantation (allo-HSCT). While cell-mediated immunity (CMI) is critical to inhibiting VZV reactivation, CMI is not routinely assessed due to a lack of reliable tests. In this study, we aimed to evaluate VZV-specific CMI among allo-HSCT recipients (n = 60) and healthy individuals (HI, n = 17) through a panel of three immune functional assays after *ex vivo* stimulation by VZV antigen: quantification of (i) IFN-γ release in the supernatants, (ii) T-cell proliferation after a 7-day stimulation of peripheral blood mononuclear cells (PBMC), and (iii) measurement of the *ifn*-*γ* mRNA gene expression level after 24 h of stimulation of a whole-blood sample. VZV responsiveness was defined according to IFN-γ release from VZV-stimulated PBMC. Upon VZV stimulation, we found that allo-HSCT recipients at a median time of 6 [5-8] months post-transplant had lower IFN-γ release (median [IQR], 0.34 [0.12–8.56] vs. 409.5 [143.9–910.2] pg/ml, *P* <.0001) and fewer proliferating T cells (0.05 [0.01–0.57] % vs. 8.74 [3.12–15.05] %, *P* <.0001) than HI. A subset of allo-HSCT recipients (VZV-responders, n = 15/57, 26%) distinguished themselves from VZV-non-responders (n = 42/57, 74%; missing data, n = 3) by higher IFN-γ release (80.45 [54.3–312.8] vs. 0.22 [0.12–0.42] pg/ml, *P* <.0001) and T-cell proliferation (2.22 [1.18–7.56] % vs. 0.002 [0.001–0.11] %, *P* <.0001), suggesting recovery of VZV-specific CMI. Interestingly, VZV responders had a significant fold increase in *ifn-γ* gene expression, whereas *ifn-γ* mRNA was not detected in whole blood of VZV-non-responders (*P* <.0001). This study is the first to suggest that measurement of *ifn-γ* gene expression in 24-h-stimulated whole blood could be an accurate test of VZV-specific CMI. The routine use of this immune functional assay to guide antiviral prophylaxis at an individual level remains to be evaluated.

## Introduction

Allogeneic hematopoietic stem cell transplantation (allo-HSCT) recipients are at increased risk of reactivation of latent varicella zoster virus (VZV) infection, with up to half of patients experiencing herpes zoster (HZ) during the first 5 years post-transplant (1–4). In addition, HZ may be more severe after allo-HSCT and exposes recipients to complications ranging from post-herpetic neuralgia to threatening disseminated disease (4–7). Although there is no immunological correlate of protection, T-cell response is thought to be the main driver of immune-mediated control of latent VZV infection (8–12). Indeed, the risk of HZ is highest during the first 24 months post-transplant, a period which corresponds to the phase of reconstitution of adaptive immunity after allo-HSCT (1–4, 13–15). Vaccination might be an interesting option to enhance VZV immunity. However, the recombinant subunit zoster vaccine (RZV) is not available in many countries while the live-attenuated VZV vaccines are contraindicated during the first 24 months post-transplant. Data regarding immunogenicity and safety of these vaccines are scarce in this setting. Pending recovery of VZV-specific immunity, complementary prophylactic strategies are thus essential to preventing VZV disease.

Currently, prevention of VZV disease in seropositive allo-HSCT recipients relies on antiviral prophylaxis with (val)acyclovir or famciclovir recommended for at least 12 months post-transplant or longer in case of protracted immune recovery (6, 16). Duration of antiviral prophylaxis may be different from a recipient to another, raising the issue of the optimal timing of discontinuation (16, 17). An informative test of VZV-specific cell-mediated immunity status could therefore help to manage VZV prophylaxis at a personalized level.

VZV-specific cell-mediated immunity can be assessed by immune functional assays (IFA) which evaluate cellular immune responses to *in vitro* stimulation by VZV antigens. These assays can be performed on peripheral blood mononuclear cells (PBMC) or whole-blood samples, and different readouts of VZV-specific cell-mediated immunity can be used, such as evaluation of lymphocyte proliferative response or quantification of interferon (IFN)-γ secretion (IFN-γ release assay, IGRA) (18–23). Nevertheless, assays on PBMC are cumbersome and time- and resource-consuming, so that they are not routinely performed. A fast, reproducible IFA monitoring VZV-specific immunity out of a small volume of whole-blood sample is of major interest to discriminate recipients having VZV-specific efficient immune response. We hypothesized that an upstream measurement of *ifn-γ* gene expression in 24-h-stimulated whole blood might correlate with VZV-specific cell-mediated immune status, as determined by standard IFA performed on PBMC. In this proof-of-concept, cross-sectional study, we determined the VZV-specific cell-mediated immune status of a cohort of adult allo-HSCT recipients at approximately 6 months post-transplant using three methods: an innovative *ifn-γ* mRNA gene expression assay from a small whole-blood sample, an automatized enzyme-linked immunosorbent IGRA, and an *ex vivo* lymphocyte proliferation assay, both performed on participant PBMC.

## Materials and Methods

### Study Population

Consecutive, adult allo-HSCT recipients transplanted at the hematology department of the Lyon University Hospital (France) and participating in the prospective, single-center “VaccHemInf” cohort study between May 2018 and August 2020 were enrolled. The VaccHemInf cohort study aims at studying vaccine response and immune reconstitution in HSCT recipients, as described elsewhere (24). Briefly, at inclusion, the demographic, hematological, and transplant-related characteristics of allo-HSCT recipients were collected. Immune reconstitution was assessed at a quantitative level (immunophenotyping of lymphocyte subsets, dosing of immunoglobulins) and at a functional level by IFA for an arbitrarily chosen subset of patients (approximately one patient in two). At our center, stem cell grafts are T-cell replete but recipients might receive antithymocyte globulin (ATG), depending on HSCT protocols. VZV-related clinical manifestations were retrospectively retrieved through medical charts. According to local procedures, antiviral prophylaxis by oral valacyclovir 500 mg bid was initiated at allo-HSCT and continued for at least 12 months. The study has been approved by a regional review board (*Comité de Protection des Personnes Sud-Est V*, Grenoble, France, number 69HCL17_0769) and is registered in ClinicalTrials.gov (NCT03659773).

Concomitantly, blood samples from healthy individuals (HI), recruited among adult donors to the Lyon (France) blood bank (*Etablissement Français du Sang*, EFS), were obtained to serve as controls. According to the EFS-standardized procedures for blood donation and to provisions of the articles R.1243–49 and following ones of the French public health code, a written non-opposition to the use of donated blood for research purposes was obtained from HI. The blood donors’ personal data were anonymized before transfer to the research laboratory. Regulatory authorizations for the handling and conservation of these samples were obtained from the regional review board (*Comité de Protection des Personnes Sud-Est II*) and the French ministry of research (*Ministère de l’Enseignement supérieur, de la Recherche et de lʼInnovation*, DC-2008–64). Blood samples from children (VZV-naïve controls) were provided as part of a study approved by the regional review board (*Comité de Protection des Personnes Sud-Est III*, 2013-011B), French Data Protection Authority (CNIL, DR-2013-354), and French Advisory Committee on Healthcare Research Data Processing (CCTIRS, 2013.223). Parents’ written consent was obtained for each participant.

### Quantification of VZV Antibodies

VZV-specific immunoglobulin (Ig)G was measured in sera by a chemiluminescence immunoassay (VZV IGG, LIAISON^®^ XL; DiaSorin, Saluggia, Italy) according to the manufacturers’ instructions. An IgG antibody titer ≥ 150 mIU/ml was considered positive (25).

### IFN-γ Release Assay and T-Cell Proliferation Measured on VZV-Stimulated Peripheral Blood Mononuclear Cells

As previously described, fresh PBMC were purified from a heparin-coated blood tube (24). Fresh PBMC were plated onto a 96-well plate at 1 × 10^5^ cells per well and incubated at 37°C and 5% of CO_2_ overnight. PBMC were then stimulated with either VZV grade 2 antigen, a gamma-irradiated antigen preparation corresponding to the supernatants of a VZV-infected cell line (MRC-5 cells; Microbix Biosystems, Mississauga, Ontario, Canada), or MRC-5 control antigen (supernatants from uninfected MRC-5 cells, CTRL) at 20 μg/l, or medium alone (non-stimulated control, NUL) for 7 days. A 3-day stimulation with phytohemagglutinin (PHA; Remel, Oxoid, Thermo Fisher Scientific, Waltham, MA, USA) at 4 µg/l served as mitogen control and medium alone (NUL) as its related negative control condition. Concentrations of stimulants, as well as length of incubation, had been determined by preliminary assays (data not shown).

At each time point, IFN-γ was measured in PBMC supernatants using the ELLA nanofluidic system (Bio-Techne, Minneapolis, MI, USA) according to the manufacturers’ instructions. All values below the lowest limit of quantification (LLOQ = 0.17 pg/ml) provided by the manufacturer were reduced to a value equal to LLOQ/√2 (= 0.12 pg/ml). Patients were classified into “VZV-responders” and “VZV-non-responders” according to the concentration of IFN-γ in supernatants from VZV-stimulated PBMC, with a positivity threshold arbitrarily fixed at twice (4.83 pg/ml) the highest secretion obtained in the control condition (2.415 pg/ml).

Pellets collected from paired wells were analyzed for T-cell proliferation using the Click-It™ EdU AF488 flow kit (C10420; Life Technologies, Carlsbad, CA, USA) to measure incorporation of 5-ethynyl-2′-deoxyuridine (EdU) according to the previously published protocol (26). Briefly, the percentage of EdU^+^ proliferating cells (among CD3^+^ cells) was obtained by flow cytometry analyses performed on a Navios flow cytometer (Beckman Coulter, Brea, CA, USA). For each experiment, a minimum of 2.5 × 10^3^ CD3^+^ cells were recorded. Data were analyzed using Kaluza software (version 1.2, Beckman Coulter). Values equal to “zero” proliferation were set to 0.01% to be plottable on a logarithmic scale.

### *ifn-γ* mRNA Gene Expression Assay on VZV-Stimulated Whole Blood

Heparinized whole blood (100 µl) was distributed into a 96-well plate. VZV antigen at 20 µg/l (VZV), medium alone (non-stimulated control, NUL), or supernatants from uninfected MRC-5 cells (negative control, CTRL) were added to a single well and incubated for 24 h at 37°C and 5% of CO_2_. Total RNA was extracted using the RNeasy Blood Mini Kit (Qiagen, Hilden, Germany) according to the manufacturer’s instructions. Briefly, the cells were harvested and lysed in RLT buffer (lysis buffer) supplemented with β-mercaptoethanol and stored at -80°C until further processing. RNA quantity and quality were determined using NanoDrop (Thermo Fisher Scientific, MA, USA). For mRNA detection, RNA was retro-transcribed using SuperScript VILO cDNA Synthesis kit (Thermo Fisher) followed by qPCR performed using commercial TaqMan probes for *ifn-γ* (Invitrogen, Carlsbad, CA, USA) and normalized using the mean of *PPIB* and *DECR1* housekeeping genes on CFX Connect Real-Time (RT)-PCR Detection System (Bio-Rad, Hercules, CA, USA). The relative differential expression of the *ifn-γ* gene was expressed as a fold change using the 2-ΔΔCt method (27). The first ΔCT is the difference in threshold cycle between the *ifn-γ* gene and the geometric mean of housekeeping in all conditions (VZV, CTRL, or NUL). ΔΔCT is the difference of *ifn-γ* gene ΔCT obtained in stimulated conditions (VZV or CTRL) compared to the unstimulated control condition (NUL).

### Post-Transplant T-Cell Immunophenotyping

A large panel of T-cell membrane markers was measured by flow cytometry (Beckman Coulter, Villepinte, France) on whole blood. Naïve (CD45RA^+^CCR7^+^) CD4^+^ and CD8^+^ T-cells, central memory (CD45RA^-^CCR7^+^) CD4^+^ and CD8^+^ T-cells, effector memory (CD45RA^-^CCR7^-^) CD4^+^ and CD8^+^ T-cells, and differentiated memory (CD45RA^+^CCR7^-^) CD4^+^ and CD8^+^ T-cells were counted (cells/μL), in addition to routine absolute CD4^+^ and CD8^+^ T lymphocyte counts.

### Statistical Analysis

Qualitative variables were expressed as counts (percentages), and quantitative continuous data were expressed as mean and range or as median and interquartile range [IQR]. Quantitative non-gaussian data were compared using the Kruskal–Wallis test or the Mann–Whitney test, where appropriate. Qualitative data were compared between groups using Fisher’s exact test. The association between quantitative data was quantified by a non-parametric Spearman rank-correlation coefficient (95% confidence interval [CI]). Two-sided tests were performed with statistical significance defined by *P* <.05. Statistical analyses were conducted using GraphPad Prism^®^ software (version 8.3; GraphPad software, La Jolla, CA, USA) and R (version 3.4.4; R Foundation for Statistical Computing, Vienna, Austria).

## Results

### Participant Characteristics

HI (n = 17) and allo-HSCT recipients (n = 60) were included. HI and allo-HSCT recipients did not significantly differ in terms of age (median [IQR], 42 [33–51] vs. 44.5 [34–60] years, *P* = .39) and sex (sex ratio, 1.1 vs. 1.3, *P* = .79). Allo-HSCT recipients were enrolled at a median [IQR] time of 6 [5-8] months post-transplant. All HI were VZV-seropositive, 98% (n = 58/59; missing data, n = 1), and 75% (n = 45/60) of allo-HSCT recipients had positive VZV IgG titers prior to transplantation and at enrollment, respectively. Nineteen (32%) allo-HSCT recipients were on immunosuppressive therapy, and 41 (68%) had received intravenous Ig infusions at a median [IQR] time of 4 [3-6] months before inclusion (**Table 1**). VZV IgG titers at inclusion were not higher among patients having received intravenous Ig infusions (*P* = .16). Pediatric controls (n = 2) were VZV-naïve.

**Table 1:** Baseline characteristics of allogeneic hematopoietic stem cell transplantation recipients.

				n = 60
**Demographics**				
** **	Age (years), median [IQR]			44.5 [34–60]
	Male, n (%)			34 (57)
**Hematological and transplant-related characteristics, n (%)**				
	Underlying hematological disease*			
		Acute myeloid leukemia and related neoplasms		31 (52)
		Myelodysplastic syndromes		8 (13)
		Myeloproliferative neoplasms		1 (2)
** **		B-lymphoblastic leukemia/lymphoma		11 (18)
		T-lymphoblastic leukemia/lymphoma		2 (3)
		Mature neoplasms: T, NK, or B cells		3 (5)
		Hodgkin lymphoma		1 (2)
		Others		3 (5)
** **	*Donor type*			
		Matched sibling donor		18 (30)
** **		Haploidentical related donor		12 (20)
		Unrelated donor		30 (50)
** **			Fully matched	23 (38)
			HLA mismatched	7 (12)
** **	*Stem cell source*			
		Peripheral blood stem cells		48 (80)
** **		Bone marrow		11 (18)
** **		Cord blood		1 (2)
	*Conditioning regimen*			
** **		MAC		22 (37)
		RIC		38 (63)
** **		TBI		18 (30)
	*GVHD prophylaxis*			
		ATG		34 (57)
		Calcineurin inhibitors		59 (98)
		Mycophenolate mofetil		33 (55)
		Methotrexate		14 (23)
		Post-transplant cyclophosphamide		23 (38)
	*Recipient VZV status prior to transplantation* ^†^			
** **		Positive		58 (98)
		Negative		1 (2)
**Post-transplant VZV IgG titers at inclusion (mIU/mL), median [IQR]**				418.5 (151.2–1092)
**Post-transplant complications, n (%)**				
** **	Acute GvHD			42 (70)
		Grade I/II/III		28/12/2
** **	Chronic GvHD			8 (13)
		Limited/extensive		5/3
**Time from transplantation (months), median [IQR]**				6 [5-8]
**Immunophenotyping of lymphocyte subsets (cells/µL), mean (range)**				
** **	Lymphocytes (NV, 1,000–2,800/µl)			1,601 (410–4,910)
	CD3^+^ T-lymphocytes (NV, 521–1,772/µl)			887 (145–3,406)
** **	CD3^+^ CD4^+^ T-lymphocytes (NV, 336–1,126/µl)			270 (38–876)
		Naïve CD4^+^ (CD45RA^+^CCR7^+^) (NV, 121–456/µl)		30 (0–390)
** **		CM CD4^+^ (CD45RA^–^CCR7^+^) (NV, 92–341/µl)		61 (1–211)
		EM CD4^+^ (CD45RA^–^CCR7^–^) (NV, 59–321/µl)		157 (4–704)
** **		DM CD4^+^ (CD45RA^+^CCR7^–^) (NV, 11–102/µl)		22 (0–226)
	CD3^+^ CD8^+^ T-lymphocytes (NV, 125–780/µl)			580 (50–2,779)
** **		Naïve CD8^+^ (CD45^+^CCR7^+^) (NV, 86–257/µl)		39 (0–241)
		CM CD8^+^ (CD45RA^–^CCR7^+^) (NV, 19–93/µl)		18 (0–127)
** **		EM CD8^+^ (CD45RA^–^CCR7^–^) (NV, 15–162/µl)		252 (0–1,517)
		DM CD8^+^ (CD45RA^+^CCR7^–^) (NV, 39–212/µl)		276 (0–1,500)
** **	CD4^+^/CD8^+^ ratio (NV, 0.9–6)			0.97 (0.12–9.07)
	CD20^+^ B-lymphocytes (NV, 64–593/µl)			282 (14–1,439)
	NK cells (NV, 70–523/µl)			235 (30–2,023)
** **	Immunoglobulin G titers (NV, 7–16 g/l)			8.6 (2.0–20.3)
**Post-transplant immunomodulatory therapy, n (%)**				
	IS therapy at inclusion^‡^			19 (32)
** **	IVIG infusion(s)			41 (68)
	Time since last IVIG infusion (months), median [IQR]			4 [3–6]
** **	DLI			8 (13)

*Based on 2016 revisions of the World Health Organization classification of myeloid and lymphoid neoplasms (28, 29). ^†^Missing value, n = 1. ^‡^Immunosuppressive therapies included cyclosporine (n = 9), tacrolimus (n = 4), corticosteroids (n = 8), ruxolitinib (n = 3). Abbreviations: ATG, antithymocyte globulin; CM, central memory; DM, differentiated memory; DLI, donor lymphocyte infusion; EM, effector memory; GvHD, graft-versus-host disease; HLA, human leukocyte antigen; IQR, interquartile range; IS, immunosuppressive; Ig, immunoglobulin; IVIG, intravenous immunoglobulins; MAC, myeloablative conditioning; NK, natural killer; NV, normal values; RIC, reduced intensity conditioning; TBI, total body irradiation; VZV, varicella zoster virus.

### Cell-Mediated Immunity Upon Phytohemagglutinin Stimulation

First, to verify that HI and allo-HSCT recipients were able to mount a detectable immune response upon mitogen stimulation, IFN-γ in supernatants and proliferation of PHA-stimulated PBMC were determined. HI and allo-HSCT recipients developed a significantly higher IFN-γ release and percentage of EdU^+^ proliferating cells (among CD3+ cells) (*P* <.0001 for both) compared to control conditions, indicating that these tests were appropriate to assess lymphocyte functionality in the study population. However, IFN-γ release (median [IQR], 5863 [1,335–16,602] vs. 74,884 [44,574–89,385] pg/ml, *P* = .004) and proliferation capacity of T-cells (median [IQR], 23.8 [14.6–33.7] % vs. 39.9 [34–48.1] %, *P* = .02) were significantly lower in allo-HSCT recipients than in HI (**Supplementary Figure 1**). Among pediatric controls, IFN-γ release and T-cell proliferation (EdU^+^ CD3^+^ cells) upon PHA stimulation were 52.6–210.3 pg/ml and 38.5%–45.4%, respectively.

### VZV-Specific Cell-Mediated Immunity

**Figure 1:**
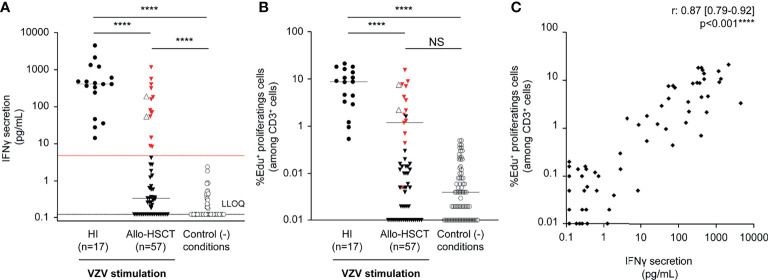
VZV-specific cell-mediated immunity according to immunoassays on peripheral blood mononuclear cells. Peripheral blood mononuclear cells isolated (1 × 10^5^ cells) from healthy individuals (HI, n = 17) and allogeneic hematopoietic stem cell transplant recipients (allo-HSCT, n = 57) were stimulated by VZV antigen (inactivated supernatants from VZV-infected MRC-5 cells, 20 µg/ml) or supernatants from VZV-uninfected MRC-5 cells (control condition) and incubated for 7 days in duplicate. **(A)** IFN-γ release (pg/ml) in the supernatants was quantified using the ELLA nanofluidic system. Bars represent median values, black dotted line represents the lower limit of quantification (LLOQ = 0.17 pg/ml), and red dotted line represents the arbitrarily defined threshold of positivity of the assay, i.e., 4.83 pg/ml, corresponding to twice the maximum value observed in the control condition. All values below LLOQ have been set at 0.12 pg/ml corresponding to LLOQ/√2. **(B)** In respective wells, T-cell proliferation is expressed as the percentage of EdU^+^ proliferating cells (among CD3^+^ cells) measured by flow cytometry after EdU^+^ incorporation and completion of the Click-it^®^. Groups were compared using Kruskal–Wallis test; ****P <.0001, NS: not significant. Red triangles represent recipients defined as VZV-responders, i.e., having recovered a VZV-specific cellular-mediated immunity according to the IFN-γ release assay. Clear triangles represent recipients who developed herpes zoster-related manifestations. **(C)** Correlation analysis between T-cell proliferation and IFN-γ release results obtained from the same well was performed using Spearman rank-correlation test; the P-values and the correlation coefficient are indicated. Data were missing for three VZV-stimulated allo-HSCT recipients.

A 7-day stimulation of PBMC with VZV antigen resulted in a significantly lower IFN-γ release in the supernatant (median [IQR], 0.34 [0.12–8.56] vs. 409.5 [143.9–910.2] pg/ml, *P* <.0001) and a significantly lower percentage of EdU^+^ proliferating cells (among CD3^+^ cells) (0.05 [0.01–0.57] % vs. 8.74 [3.12–15.05] %, *P* <.0001) among allo-HSCT recipients as compared to HI (**Figures 1A, B**). Both were highly correlated (Spearman’s correlation coefficient 0.87; 95% CI [0.79–0.92]; **Figure 1C**). Conversely, VZV-specific T-cell proliferation was not correlated with unspecific PHA-induced T-cell proliferation (Spearman’s correlation coefficient, 0.07 [95% CI, -0.2–0.34]). Of note, VZV-naïve controls did not mount any proliferative response (no EdU^+^ proliferating CD3^+^ cells: 0%–0%) and had IFN-γ release below LLOQ (0.104–0.131 pg/ml).

Results indicated that allo-HSCT recipients had an impaired VZV-specific T-cell function based on lower proliferation and IFN-γ release post-stimulation. When focusing on allo-HSCT recipients’ results, two subsets were observed and classified as VZV responders (n = 15/57, 26%) or non-responders (n = 42/57, 74%; missing data, n = 3) based on IFN-γ secretion level above the positivity threshold (**Table 2**). Among the VZV-responder group, a significantly higher IFN-γ release was observed, as compared to the VZV-non-responder group (median [IQR], 80.45 [54.3–312.8] vs. 0.22 [0.12–0.42] pg/ml, *P* <.0001). The IFN-γ release among the VZV-responder group was lower than among HI (median [IQR], 409.5 [143.9–910.2] pg/ml), but the difference did not reach statistical significance (*P* = .64). Of note, similar observations were made for the VZV-specific proliferative response, with a significant higher proliferation capacity in the VZV-responder group, as compared to the VZV-non-responder group (median [IQR], 2.22 [1.18–7.56] % vs. 0.002 [0.001–0.11] %, *P* <.0001). Proliferative response among the VZV-responder group was lower than in HI without reaching statistical significance (median [IQR], 8.74 [3.12–15.05] %, *P* = .76).

**Table 2:** VZV-specific immune responses of allogeneic hematopoietic stem cell transplantation recipients.

		VZV non-responders	VZV responders	*P*
		(n = 42)*	(n = 15)	
**7-day VZV-stimulated PBMC, median [IQR]**		** **	** **	*** ***
	IFN-γ release (pg/mL)	0.22 [0.12–0.42]	80.45 [54.3–312.8]	**<.0001**
	EdU^+^ proliferating cells among CD3^+^ cells (%)	0.002 [0.001–0.11]	2.22 [1.18–7.56]	**<.0001**
**24-h VZV-stimulated whole blood** ^†^, **median [IQR]**				
** **	*ifn*-*γ* gene expression (fold change)	0.85 [0.55–1.44]	12.99 [5.30–79.79]	**<.0001**
**Immunophenotyping of lymphocytes and subsets (cells/µL), median [IQR]**				
	ALC	1,090 [710–2,460]	1,590 [1,000–2,130]	.421
** **	T CD4^+^	232.5 [115.5–385.5]	287 [220.5–329]	.319
	T CD4^+^ N	12.5 [4.75–22.25]	33 [14.5–38]	**.043**
	T CD4^+^ CM	40 [17–80]	81 [65.5–114]	**.010**
	T CD4^+^ EM	115 [80–208]	157 [116.5–192.5]	.459
	T CD4^+^ DM	5.5 [1.75–38.75]	3.5 [0–5.75]	.106
	T CD8^+^	367.5 [218.75–879.25]	364 [237.5–486]	.921
	T CD8^+^ N	16.5 [9–50.5]	44 [24–70.5]	**.048**
	T CD8^+^ CM	10 [2–14.5]	16 [7–30]	.125
	T CD8^+^ EM	160 [69.5–282.75]	157 [108.5–303]	.490
	T CD8^+^ DM	225 [60–332]	102 [64.5–140]	.287
	NK cells	157 [114–137]	146 [99.5–194]	0.39
**Post-transplant VZV IgG titers at inclusion (mIU/mL), median [IQR]**		383.1 [141.1–664.2]	1382 [494.4–1577]	**.005**
**Selected clinical variables, n (%)**				
	AML	20 (48)	9 (60)	.715
	ATG	24 (57)	9 (60)	>.999
	DLI	5 (12)	3 (20)	.422
	Chronic GvHD	5 (12)	3 (20)	.422
	IS therapy^‡^	15 (36)	4 (27)	.751

Peripheral blood mononuclear cells (PBMC) (1 × 10^5^ cells) and whole blood from allo-HSCT recipients (n = 57) were stimulated by VZV antigen (inactivated supernatants from VZV-infected MRC-5 cells, 20 µg/ml) or supernatants from VZV-uninfected MRC-5 cells (control condition). IFN-γ release (pg/ml) and the percentage of EdU^+^ proliferating cells (among CD3^+^ cells) were measured after a 7-day VZV stimulation of PBMC by immunoassay (ELLA nanofluidic system) or flow cytometry, respectively. VZV responders were identified based on IFN-γ release strictly above 4.83 pg/ml, corresponding to twice the maximum value observed in the control condition. The ifn-γ gene expression level was measured by qPCR in a whole blood sample after 24 h of VZV stimulation. The relative differential expression between VZV-stimulated and non-stimulated conditions is given as the fold change. Immunophenotyping of lymphocyte subsets was performed by flow cytometry on whole blood. Groups were compared using the Mann–Whitney U test or the Fisher’s exact test, where appropriate; P-values <.05 were considered statistically significant and are highlighted in bold. *Data were missing for three allo-HSCT recipients. †Data were missing for three allo-HSCT recipients. ^‡^Immunosuppressive therapy: cyclosporine, tacrolimus, corticosteroids, ruxolitinib. Abbreviations: ALC, absolute lymphocyte count; AML, acute myeloid leukemia; ATG, antithymocyte globulin; CM, central memory; DM, differentiated memory; DLI, donor lymphocyte infusion; EdU, 5-ethynyl-2′-deoxyuridine; EM, effector memory; GvHD, graft-versus-host disease; IFN-γ, interferon-γ; IgG, immunoglobulin G; IQR, interquartile range; IS, immunosuppressive; N, naïve; VZV, varicella zoster virus.

Interestingly, 13/15 (87%) of VZV responders had a positive VZV-specific humoral response, as depicted by higher IgG titers (median [IQR], 1382 [494.4–1577] mIU/ml), as compared to the VZV-non-responder group, where 29/42 (69%) had a positive titer (median [IQR], 383.1 [141.1–664.2] mIU/ml, *P* = .005). Altogether, these results suggest an ongoing recovery of VZV-specific immunity in the VZV-responder group. Only 2/15 (13%) had reported HZ-compatible clinical manifestations at 4 and 5 months post-transplant, respectively, while none in the VZV-non-responder group.

### *ifn-γ* mRNA Gene Expression in Response to Whole Blood VZV Antigen Stimulation

**Figure 2:**
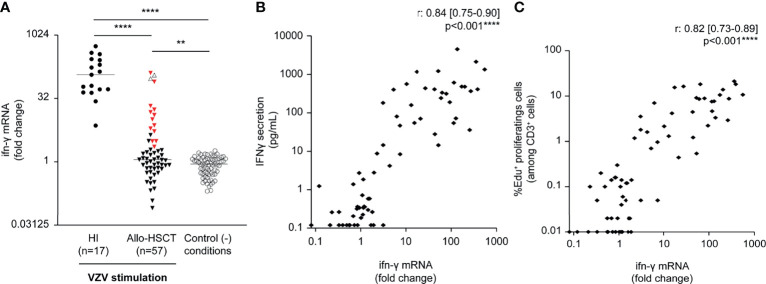
*Ex vivo* whole blood VZV-stimulation and correlations between VZV-specific immune functional assays. Whole blood (100 µl) from healthy individuals (HI, n = 17) and allogeneic hematopoietic stem cell transplant recipients (allo-HSCT, n = 57) was stimulated 24 h by VZV antigen (inactivated supernatants from VZV-infected MRC-5 cells, 20 µg/ml) or supernatants from VZV-uninfected MRC-5 cells (control condition). **(A)** The relative quantification of *ifn-γ* gene expression levels is presented as fold change. Red triangles represent recipients defined as VZV responders, according to IFN-γ release in supernatants from VZV-stimulated peripheral blood mononuclear cells (see **Figure 1**). Clear triangles represent recipients who developed herpes zoster-related manifestations. Bars represent median and *P*-values were obtained using the Kruskal–Wallis test; ** *P* <.01, ****P <.0001. **(B)** Correlation between *ifn-γ* mRNA gene expression (fold change) and IFN-γ release in supernatants from VZV-stimulated PBMC quantified using the ELLA nanofluidic system, using Spearman rank-correlation test. **(C)** Correlation between *ifn-γ* mRNA gene expression (fold change) and percentage of EdU^+^ cells (among CD3^+^) measured by flow cytometry, using Spearman rank-correlation test. Data were missing for three VZV-stimulated recipients.

To investigate the VZV-specific immune response at the transcriptional level, the relative differential expression (fold change) of *ifn-γ* mRNA at 24 h in VZV-stimulated whole blood was compared to that found in non-stimulated conditions. The VZV responders according to IFN-γ secretion from PBMC (n = 15) had a significant fold increase in *ifn-γ* gene expression in whole blood (median [IQR], 12.99 [5.3–79.79]), whereas the relative expression of *ifn-γ* mRNA was not detected for other allo-HSCT recipients (median [IQR], 0.85 [0.55–1.44], *P* <.0001) (**Figure 2A**). Moreover, *ifn-γ* gene expression in allo-HSCT recipients and HI was significantly correlated with IFN-γ release and percentage of EdU^+^ proliferating cells (among CD3^+^ cells) (Spearman’s correlation coefficients, 0.84, 95% CI [0.75–0.9] and 0.82, 95% CI [0.73–0.89], respectively; **Figures 2B, C**). Overall, these results indicate a consistency between upstream *ifn-γ* gene expression at 24 h in VZV-stimulated whole blood and downstream lymphocyte proliferative and IFN-γ-releasing capacities of PBMC at day 7 in response to VZV antigen. In VZV-naïve controls, *ifn-γ* mRNA was not significantly expressed after VZV stimulation (fold change: 0.66–1.87). Overall, results of VZV-specific IFA in VZV-naïve pediatric controls confirmed the specificity of these assays.

### Post-Transplant T-Cell Immunophenotyping and Correlation With IFN-γ Release

The correlation between VZV-induced IFN-γ release and various T-cell subsets in allo-HSCT recipients was investigated. Although the median absolute CD4^+^ T-cell count was lower than normal values (**Table 1**), the IFN-γ release in response to VZV antigen was positively correlated with absolute counts of naïve (CD45RA^+^CCR7^+^) and central memory (CD45RA^-^CCR7^+^) CD4^+^ T-cells, as well as of naïve (CD45RA^+^CCR7^+^) CD8^+^ T-cells (Spearman rank-correlation coefficients, r = 0.35 (95% CI [0.08–0.57]), 0.28 (95% CI [0.02–0.52]) and 0.36 (95% CI [0.10–0.58]), respectively). When comparing VZV responders and non-responders, median naïve and central memory CD4^+^ and naïve CD8^+^ T-cell counts were significantly higher in the VZV-responder group (*P* = .043, *P* = .01, *P* = .048, respectively). Of note, no associations were observed between VZV responsiveness and clinical determinants (**Table 2**).

## Discussion

This study provides insights into VZV-specific immune reconstitution after allo-HSCT. We found that 15/57 (26%) of allo-HSCT recipients had recovered a VZV-specific IFN-γ response by 6 months post-transplant, in the absence of any HZ-related clinical event for the majority of them. Interestingly, the measurement of *ifn-γ* gene expression by qPCR at 24 h on a small-volume whole-blood sample could be as effective and less time-consuming than standard IFA based on the quantification of lymphocyte proliferation and IFN-γ release upon VZV antigen stimulation to identify allo-HSCT recipients that have recovered VZV-specific cell-mediated immunity. In addition, we found that naïve (CD45RA^+^CCR7^+^) T-cell counts and central memory (CD45RA^-^CCR7^+^) CD4^+^ T-cell counts correlated with the reconstitution of the VZV-specific immune repertoire.

Reconstitution of VZV-specific immunity following allo-HSCT is mostly driven by endogenous VZV reexposure, rather than by exogenous reinfection, and can occur with or without clinical manifestations and despite antiviral prophylaxis (20, 21, 30–32). Subclinical VZV reactivations are well described in immunocompromised patients (18–20, 32). In the present study, the majority (13/15, 87%) of VZV responders had not presented any HZ-compatible manifestation, although *in vitro* assays showed detectable VZV-specific immune cell responses. Relative to the entire cohort of allo-HSCT recipients, this proportion of subclinical VZV reactivations (13/60, 22%) is overall consistent with previous studies that reported 26% to 41% of subclinical VZV reactivations among adult bone marrow transplant recipients (20, 33).

Importantly, unresponsiveness to VZV was not associated with the receipt of immunosuppressive treatment, suggesting that the process of immune reconstitution *per se* is individual. IFN*-*γ release in response to VZV stimulation was correlated with naïve CD4^+^ and CD8^+^ and central memory CD4^+^ T-cell subsets despite low total CD4^+^ T-cell absolute counts. This suggests that thymopoiesis contributes to the reconstitution of cognate cell-mediated immune responses and an antiviral-specific T-cell repertoire (34), rather than survival of host cells or expansion of donor-derived T-cells. Therefore, detection of VZV-specific cell-mediated immunity might be a surrogate marker of the reconstitution of a thymus-dependent, functional T-cell pool after allo-HSCT, although quantitative naïve T-cell assessment did not strongly correlate with functional response. Importantly, the total CD4^+^ T-cell count did not discriminate VZV responders from VZV non-responders: more than half of VZV non-responders had a total CD4^+^ T-cell count above 200/µl, a threshold of the immunosuppressive state validated in the context of human immunodeficiency virus infection, but that many clinicians use to guide antimicrobial prophylaxis strategies in other settings of immunodepression. Furthermore, no hematological or transplant-related characteristic was associated with VZV responsiveness.

Humoral immunity to VZV, measured by IgG antibody titers, is known not to correlate with immune protection, and infusions of blood-derived products, in particular intravenous Ig, can lead to non-interpretable results (8, 20, 22, 35). Consistently, 29/42 (69%) of VZV non-responders had positive VZV IgG values in this study. Different IFA have been proposed to quantify VZV-specific cell-mediated immunity, but there is no validated “gold standard.” VZV-specific T-cell proliferation assays, performed on PBMC or whole blood (5, 20, 22, 36, 37), have several limitations. The first relates to the various origins of IFN-γ production. The correlation between VZV-specific IFN-γ release and T-cell subsets suggests that IFN-γ is mainly secreted by T-cells. To gain the detection sensitivity of VZV-responsive T-cells at the single-cell level, the IFN-γ enzyme-linked immunosorbent spot (ELISPOT) assay has been proposed (9, 31, 32, 38). However, in addition to being labor intensive because of the multiple experimental steps from PBMC preparation to spot detection, enumeration of IFN-γ spot-forming cells proves to be difficult. The second limit relates to the threshold of detection of IFN-γ. Others have reported that IFN-γ release in the supernatant of whole blood incubated with live VZV was not detected before 48 h and peaked at 72 h (22). Consequently, to test the VZV status more rapidly, we formulated the hypothesis that measuring upstream *ifn-γ* gene expression in 24-h-stimulated whole blood would predict the VZV status as effectively as downstream synthetized IFN-γ protein at day 7. qPCR on whole blood is relatively simple to perform, in comparison to various cell-based IFA. To the best of our knowledge, this is the first study assessing the *ifn-γ* gene expression level as a marker of VZV-specific cell-mediated immunity. As PCR techniques are widely used during routine post-transplant care, the implementation of the VZV-specific *ifn-γ* gene expression assay on whole blood at a lab experienced in the follow-up of allo-HSCT recipients should be relatively easy. The main advantages of PCR assays are their time- and resource-sparing nature, their relative technical ease, and their reproducibility.

Taken together and considering that one quarter of allo-HSCT recipients had detectable VZV-specific cell-mediated immunity by the time of study initiation (6 months post-transplant), measuring the VZV-specific *ifn-γ* gene expression level in whole blood once every trimester after allo-HSCT could help to monitor kinetics of specific immune reconstitution. If assumed as a surrogate marker of VZV-specific immunity, this assay, combined to T-cell immunophenotyping, could ultimately guide antiviral prophylaxis in a personalized manner. This would provide the basis for an interventional, randomized, controlled trial vs. standard of care to test the hypothesis that it is safe to stop antiviral prophylaxis based on a personalized approach using the *ifn-γ* gene expression assay. The primary endpoint would be the absence of HZ reactivation following prophylaxis interruption. An earlier interruption of antiviral prophylaxis could avoid overtreatment and emergence of HSV cross-resistance to acyclovir and limit compliance issues. Other useful applications of the *ifn-γ* gene expression assay could be proposed such as determining VZV-specific immunity status in other patient groups at risk of VZV reactivation (i.e., solid organ transplant recipients, multiple myeloma patients, elderly people) or evaluating cellular response to VZV vaccination.

Beyond antiviral prophylaxis, VZV-related disease can be prevented by vaccination. The inactivated RZV has demonstrated a clinical efficacy of 68.2% to prevent HZ after autologous HSCT and has been recently shown in a prospective cohort study to be safe after allo-HSCT, although immunogenicity data in this setting are lacking (39, 40). The RZV has been recently recommended to immunocompromised adults, including allo-HSCT recipients, in the USA, but is not currently available in France (41).

This study has limitations. The results of this proof-of-concept study require a validation cohort. We report VZV-specific immunity at a single time point (approximately 6 months post-transplant, due to the design of the VaccHemInf study which aims to study response to the inactivated vaccines which are recommended in this timeframe). The time of 6-month post-transplant corresponds to a pivotal period, when allo-HSCT recipients are usually off immunosuppressive therapy and when immune reconstitution is well underway from a quantitative point of view. We did not assess in this study kinetics of antiviral immune reconstitution. Due to the high seroprevalence of VZV (>95%) among adults in France, the specificity of VZV IFA was confirmed on pediatric samples (42, 43). The choice of VZV antigen (supernatants from VZV-infected MRC-5 cells, inactivated by gamma irradiation), although widely used, can be criticized due to cross-reactivity with other herpesvirus, especially herpes simplex virus-1, as previously reported *in vivo* (44, 45). Peptides based on VZV glycoprotein gE and immediate-early 63 might be more suitable antigens. It would also have been of interest to compare the assay with the intracellular cytokine staining method or the histocompatibility complex-tetramer technology such as previously described for CMV reactivation (46, 47); similarly, for thymopoiesis after allo-HSCT, quantitative assessment of T-cell receptor rearrangement excision circles (TRECs) could have been used as an informative surrogate marker for recipients, as previously described in the setting of viral reactivation (48).

In conclusion, as the management of immunocompromised individuals evolves to increasingly personalized agendas, measurement of VZV-specific cell-mediated immunity by a fast (24-h) *ifn-γ* gene expression assay from a whole-blood sample and immunophenotyping of T-cell subsets could help to monitor antiviral immune repertoire reconstitution after allo-HSCT and ultimately enable a more rationale use of antiviral prophylaxis.

## Data Availability Statement

The datasets presented in this article are not readily available because no patient-level data can be made available, in accordance with data protection laws. Upon reasonable request, aggregate data with different levels of resolution may be possible to provide. Requests to access the datasets should be directed to florence.ader@chu-lyon.fr.

## Ethics Statement

The studies involving human participants were reviewed and approved by Comité de Protection des Personnes Sud-Est V, Grenoble, France (69HCL17_0769). The patients/participants provided their written informed consent to participate in this study.

## Author Contributions

MB contributed to the conception and design of the study and to the acquisition and interpretation of the data and drafted the manuscript. AC and WM contributed to the acquisition and interpretation of the data and drafted the manuscript. CR-S and EF performed the VZV serology assays and contributed to the revision of the paper for important intellectual content. BR provided pediatric control samples and contributed to the revision of the paper for important intellectual content. FVa, VA, HL-W, HG, and KB-P contributed to the interpretation of the data and to the revision of the paper for important intellectual content. FVe contributed to the acquisition of the data of immune functional assays, to the interpretation of the data, and to the revision of the paper for important intellectual content. ST-A contributed to the conception and design of the study, to the acquisition and interpretation of the data, and to the revision of the paper for important intellectual content. FA conceived and designed the study, participated in the analyses and interpretation of the data, and drafted the manuscript. All authors have read and have approved the final version of the manuscript.

## Funding

This research was funded by an internal grant from the Hospices Civils de Lyon (Appel d’Offre Jeune Chercheur 2018, to AC) and by the Région Auvergne-Rhône-Alpes (Pack Ambition Recherche 2019, to FA).

## Conflict of Interest

WM has a PhD grant CIFRE 2019 (conventions industrielles de formation par la recherche, Ministère de l’Enseignement supérieur, de la Recherche et de l’Innovation, Paris, France) half-funded by Lyon University and half-funded by bioMerieux SA. KB-P is an employee of bioMérieux SA, an *in vitro* diagnostic company.

The remaining authors declare that the research was conducted in the absence of any commercial or financial relationships that could be constructed as a potential conflict of interest.

## Publisher’s Note

All claims expressed in this article are solely those of the authors and do not necessarily represent those of their affiliated organizations, or those of the publisher, the editors and the reviewers. Any product that may be evaluated in this article, or claim that may be made by its manufacturer, is not guaranteed or endorsed by the publisher.
