# Supplementary material for: A Simple-to-Perform ifn-γ mRNA Gene Expression Assay on Whole Blood Accurately Appraises Varicella Zoster Virus-Specific Cell-Mediated Immunity After Allogeneic Hematopoietic Stem Cell Transplantation
Source: Front Immunol. 2022 Jul 27;13:919806. doi: 10.3389/fimmu.2022.919806 (PMC9363621; doi:10.3389/fimmu.2022.919806)
Supplement: Supplementary file 1 [file Presentation_1.pdf]

Supplementary Material

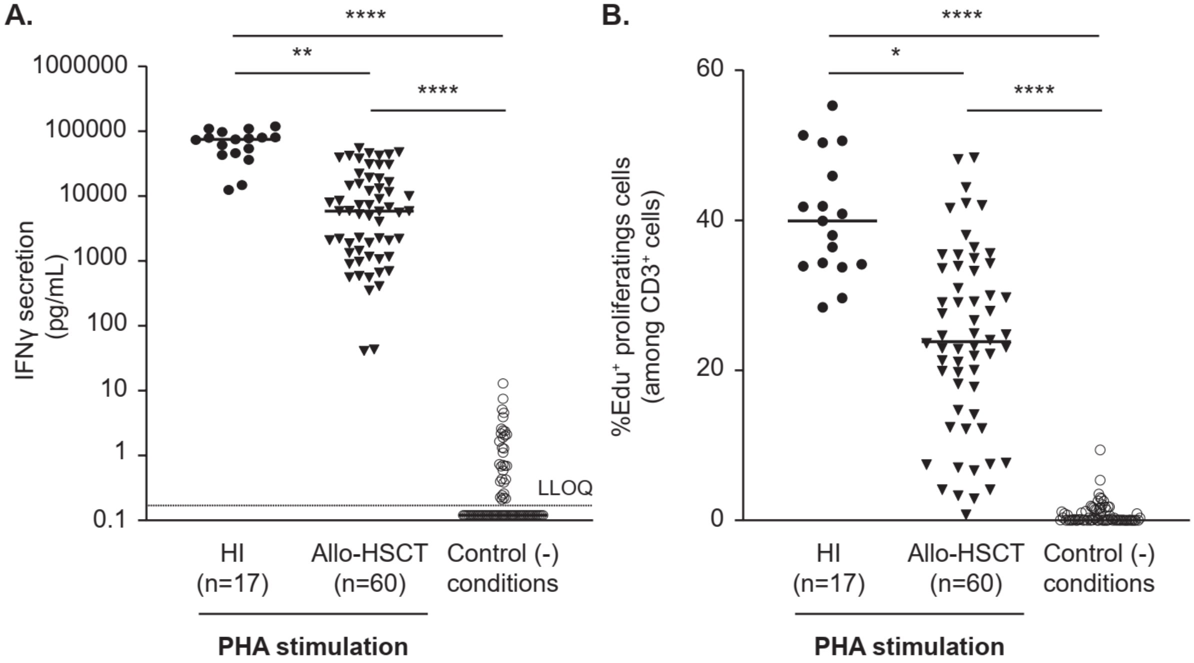

Supplementary Figure 1. PHA-induced T-cell proliferation and IFN-γ release

Peripheral blood mononuclear cells isolated (1 x 10^5 cells) from healthy individuals (HI, n=17) and allogeneic hematopoietic stem cell transplant recipients (allo-HSCT, n=60) were stimulated by phytohemagglutinin (PHA) or negative control conditions (medium) and incubated for 3 days. (A) IFN-γ release (pg/ml) in the supernatants was quantified using the ELLA nanofluidic system. Bars represent median values, black dotted line represents the lower limit of quantification (LLOQ = 0.17 pg/ml). All values below LLOQ have been set at 0.12 pg/ml corresponding to LLOQ/√2. (B) In respective wells, T-cell proliferation is expressed as the percentage of EdU^+ proliferating cells (among $CD3^+$ cells) measured by flow cytometry after EdU^+ incorporation and completion of the Click-it®. Groups were compared using Kruskal-Wallis test; * $P < .05$, ** $P < .01$, **** $P < .0001$. Data were missing for one PHA-stimulated allo-HSCT recipient.
